# Studies on Inhibition of Respiratory Cytochrome *bc*_1_ Complex by the Fungicide Pyrimorph Suggest a Novel Inhibitory Mechanism

**DOI:** 10.1371/journal.pone.0093765

**Published:** 2014-04-03

**Authors:** Yu-Mei Xiao, Lothar Esser, Fei Zhou, Chang Li, Yi-Hui Zhou, Chang-An Yu, Zhao-Hai Qin, Di Xia

**Affiliations:** 1 Department of Applied Chemistry, China Agricultural University, Beijing, China; 2 Laboratory of Cell Biology, Center for Cancer Research, National Cancer Institute, NIH, Bethesda, Maryland, United States of America; 3 Department of Biochemistry and Molecular Biology, Oklahoma State University, Stillwater, Oklahoma, United States of America; University of Illinois at Chicago, United States of America

## Abstract

The respiratory chain cytochrome *bc*_1_ complex (cyt *bc*_1_) is a major target of numerous antibiotics and fungicides. All cyt *bc*_1_ inhibitors act on either the ubiquinol oxidation (Q_P_) or ubiquinone reduction (Q_N_) site. The primary cause of resistance to *bc*_1_ inhibitors is target site mutations, creating a need for novel agents that act on alternative sites within the cyt *bc*_1_ to overcome resistance. Pyrimorph, a synthetic fungicide, inhibits the growth of a broad range of plant pathogenic fungi, though little is known concerning its mechanism of action. In this study, using isolated mitochondria from pathogenic fungus *Phytophthora capsici,* we show that pyrimorph blocks mitochondrial electron transport by affecting the function of cyt *bc*_1_. Indeed, pyrimorph inhibits the activities of both purified 11-subunit mitochondrial and 4-subunit bacterial *bc*_1_ with IC_50_ values of 85.0 μM and 69.2 μM, respectively, indicating that it targets the essential subunits of cyt *bc*_1_ complexes. Using an array of biochemical and spectral methods, we show that pyrimorph acts on an area near the Q_P_ site and falls into the category of a mixed-type, noncompetitive inhibitor with respect to the substrate ubiquinol. *In silico* molecular docking of pyrimorph to cyt *b* from mammalian and bacterial sources also suggests that pyrimorph binds in the vicinity of the quinol oxidation site.

## Introduction

The cytochrome *bc*_1_ complex (cyt *bc*_1_, also known as ubiquinone:cyt *c* oxidoreductase, Complex III or *bc*_1_) is a central component of the cellular respiratory chain of mitochondria. It catalyzes the reaction of electron transfer (ET) from ubiquinol to cyt *c* and couples this reaction to proton translocation across the mitochondrial inner membrane, contributing to the cross-membrane proton motive force essential for cellular functions such as ATP synthesis [1], [2]. The indispensible function of cyt *bc*_1_ in cellular energy metabolism makes it a prime target for numerous natural and synthetic antibiotics. More than 20 synthetic fungicides targeting cyt *bc*_1_ are in widespread use in agriculture with an annual sale exceeding $2.7 billion [3].

All cyt *bc*_1_ inhibitors target either the ubiquinol oxidation site (Q_P_ or Q_o_) or the ubiquinone reduction site (Q_N_ or Q_i_), which are defined by the Q-cycle mechanism of cyt *bc*_1_ function [4], [5]. Despite variations in subunit compositions of *bc*_1_ from various organisms, only three subunits are essential for ET-coupled proton translocation function: they are cyt *b*, cyt *c*_1_ and the iron-sulfur protein (ISP). The cyt *b* subunit contains two *b*-type hemes (*b*_L_ and *b*_H_), the cyt *c*_1_ subunit has a *c*-type heme, and the ISP possesses a 2Fe-2S cluster. Both active sites are located within the cyt *b* subunit, as demonstrated by crystallographic studies of mitochondrial and bacterial *bc*_1_ complexes [6]–[12]. Resistance to known cyt *bc*_1_ fungicides has been reported at an alarming rate, rendering many of these reagents ineffective. Most common mechanisms of resistance involve target site mutations and corresponding strategies to overcome drug resistance have been proposed [13]. Developing new agents targeting areas outside the Q_P_ and Q_N_ sites of cyt *bc*_1_ is most attractive primarily because the new compounds presumably are able to circumvent existing fungal resistance.

Pyrimorph, (Z)-3-[(2-chloropyridine-4-yl)-3-(4-*tert*-butylphenyl)-acryloyl] morpholine, is a novel systemic antifungal agent that belongs to the family of carboxylic acid amide (CAA) fungicides [14], whose members include mandipropamid, dimethomorph, flumorph, and valinine derivatives. Pyrimorph exhibits excellent activity inhibiting mycelial growth of the fungal species *Phytophthora infestans*, *Phytophthora capsici,* and *Rhizoctonia solani* and is able to suppress zoosporangia germination of *Pseudoperonospora cubensis* with EC_50_ values in the range between 1.3 and 13.5 μM [15]. The *in vitro* sensitivities of various asexual stages of *Peronophythora litchii* to pyrimorph were studied with four single-sporangium isolates, showing high sensitivity at the stage of mycelial growth with an EC_50_ of 0.3 μM [16].

Although pyrimorph is currently in use to control various fungal pathogens [15]–[17], its functional mechanism has remained unclear. The presence of a common CAA moiety has led to the suggestion that pyrimorph may work in a fashion similar to that of other CAA-type fungicides [18]. One CAA member, mandipropamid, was shown to target the pathway of cell wall synthesis by inhibiting the CesA3 cellulose synthases [19]. However, treatment of fungal pathogens with pyrimorph appeared to affect multiple cellular pathways, including, but not limited to, those of cellular energy metabolism and cell wall biosynthesis, either directly or indirectly [20]. Indeed, a recent report has correlated the pyrimorph resistance phenotype in *P. capsici* with mutations in the CesA3 gene [21].

Other mechanisms of pyrimorph action have yet to be investigated. In particular, its potential interference with cellular respiratory chain components leading to reduced ATP synthesis appears to be a reasonable hypothesis for the observed inhibitory effects on energy demanding processes such as mycelial growth and cytospore germination of fungi. Here, we report the effects of pyrimorph on electron flow through the isolated fungal mitochondrial respiratory chain and the identification of the cyt *bc*_1_ complex as pyrimorph’s primary target. Kinetic experiments suggest that the mode of pyrimorph inhibition is to interfere with substrate access to the ubiquinol oxidation site but in a way that differs from other *bc*_1_ inhibitors, suggesting a novel mode of inhibitory mechanism.

## Materials and Methods

The pyrimorph used in all experiments was synthesized in our laboratory. Dimethomorph was a gift from Jiangshu Frey Chemical Co. Ltd. (Jiangshu Province, China). Cyt *c* (from horse heart, type III) was purchased from Sigma-Aldrich (St. Louis, MI). 2,3-dimethoxy-5-methyl-6-(10-bromodecyl)-1,4-benzoquinol (Q_0_C_10_BrH_2_) was prepared as previously reported [22]. N-dodecyl-*β*-D-maltoside (β-DDM) and N-octyl-β-D-glucoside (β-OG) were purchased from Affymetrix (Santa Clara, CA). All other chemicals were purchased and are of the highest grade possible.

### Preparation of Light Mitochondria from *Phytophthora capsici*

Light mitochondrial fraction were prepared from cultured mycelia from laboratory strain *Phytophthora capsici Leonia* (*P. capsici*), which was grown in CA liquid medium (8% carrot juice and 2% glucose) for 5 days in the dark at 25°C [23]. 10 g mycelia (fresh weight) were washed with 0.6 M mannitol solution and ground up for 5 minutes with an ice-cold mortar and pestle in 100 ml buffer A containing 10 mM MOPS•KOH, pH 7.1, 0.3 M mannitol, 1 mM EDTA and 0.1% (w/v) bovine serum albumin (BSA) and 30 g of sea sand. The homogenate was centrifuged at 3,200×g for 10 min at 4°C and the supernatant was further centrifuged at 12,000×g for 30 min. The precipitate, light mitochondrial fraction, was resuspended and washed with 20 ml buffer B containing 10 mM MOPS•KOH, pH 7.1, 0.25 M sucrose and 1 mM EDTA and pelleted again by centrifugation at 12,000×g for 20 min at 4°C. The mitochondrial preparation was resuspended in buffer A and the protein concentration was adjusted to 0.1 mg/ml.

### Inhibition of the ET Activity of *P. capsici* Mitochondria by Pyrimorph

The activities of mitochondrial respiratory chain components were assayed using the Mitochondria Complex Activity Assay Kit (Genmed Scientifics, Inc. USA, Wilmington, DE) following manufacturer’s instruction. Briefly, Complex I activity was measured by following the oxidation of NADH by monitoring the decrease in absorbance difference between 340 nm and 380 nm. The reaction mixture (1 ml) consisted of 50 mM potassium phosphate buffer, pH 7.6, 0.25 mM NADH and 50 mM decylubiquinone as the electron acceptor. Crude mitochondria (200 μg protein) were added to start the reaction. Complex II activity was estimated as the rate of reduction of ubiquinone to ubiquinol by succinate, which can be followed by the secondary reduction of 2,6-dichlorophenolindophenol (DCPIP) as the ubiquinol forms. The reaction mixture (1 ml) contained 50 mM potassium phosphate buffer, pH 7.6, 20 mM succinate, 1.0 mM EDTA, 0.05 mM DCPIP and 3 mM NaN_3_, and 50 mM decylubiquinone. Crude mitochondria (65 μg) were added to initiate the reaction and the decrease in absorbance at 600 nm was followed as DCPIP becomes reduced. Complex III activity was assayed by following the increase in absorbance at 550 nm as cyt *c* becomes reduced using decylubiquinol as an electron donor. Here, the reaction mixture (1 ml) consisted of 50 mM potassium phosphate buffer, pH 7.6, 0.1% BSA, 0.1 mM EDTA, 60 mM oxidized cyt *c*, and 150 μM decylubiquinol. Crude mitochondria (10 μg protein) were then added to initiate the reaction.

### Purification of Cyt *bc*_1_ Complexes from Beef Heart and Photosynthetic Bacterium *Rhodobacter sphaeroides*

Bovine heart mitochondrial *bc*_1_ (*Btbc*_1_) complex was prepared starting from highly purified succinate-cyt *c* reductase, as previously reported [24]. The *bc*_1_ particles were solubilized by deoxycholate and contaminants were removed by a 15-step ammonium acetate fractionation. The purified *bc*_1_ complex was recovered in the oxidized state from the precipitates formed between 18.5% and 33.5% ammonium acetate saturation. The final product was dissolved in 50 mM Tris•HCl buffer, pH 7.8, containing 0.66 M sucrose resulting in a stock solution with a protein concentration of 30 mg/ml, which was stored at −80°C. The concentrations of cyt *b* and *c*_1_ were determined spectroscopically using millimolar extinction coefficients of 28.5 and 17.5 mM^−1^ cm^−1^ for cyt *b* and *c*_1_, respectively.

To prepare cyt *bc*_1_ complex from the photosynthetic bacterium *R. sphaeroides (Rsbc*_1_ *)*, *R. sphaeroides* strain BC17 cells bearing the pRKD418-*fbc*FBC_6H_Q plasmid [25] were grown photosynthetically at 30°C in an enriched Sistrom medium containing 5 mM glutamate and 0.2% casamino acids [26]. The growth was monitored by measuring the OD_600_ value every 3**–**5 h. Cells were transferred to a larger batch or harvested when OD_600_ reached 1.8**–**2.0. Chromatophore membranes were prepared from BC17 cells as described previously [27] and stored at a very high concentration in the presence of 20% glycerol at **−**80°C. To purify the hexahistidine-tagged *Rsbc*_1_ complex, the freshly prepared chromatophores or frozen chromatophores thawed on ice were adjusted to a cyt *b* concentration of 25 μM with a solubilization buffer containing 50 mM Tris•HCl, pH 8.0 at 4°C, and 1 mM MgSO_4_. 10% (w/v) β-DDM was added to the chromatophore suspension to a final concentration of 0.56 mg detergent/nmole of cyt *b* followed by addition of 4M NaCl solution to a final concentration of 0.1 M. After stirring on ice for 1 hour, the admixture was centrifuged at 220,000×g for 90 minutes; the supernatant was collected and diluted with equal volume of the solubilization buffer followed by passing through a Ni-NTA agarose column (100 nmole of cyt *b*/ml of resin) pre-equilibrated with two volumes of the solubilization buffer. After loading, the column was washed sequentially with the following buffers until the absence of greenish color in effluent was reached: washing buffer (50 mM Tris•HCl, pH 8.0 at 4°C, containing 100 mM NaCl) +0.01% β-DDM; washing buffer +0.01% β-DDM and 5 mM histidine; washing buffer +0.5% β-OG; washing buffer +0.5% β-OG and 5 mM histidine. The cyt *bc*_1_ complex was eluted with the washing buffer +0.5% β-OG and 200 mM histidine. Pure fractions were combined and concentrated by Centriprep-30 concentrator. Glycerol was added to a final concentration of 10% before storage at −80°C.

### Measurement of *bc*_1_ Activity and its Inhibition (IC_50_) by Various Inhibitors

The activities of isolated cyt *bc*_1_ complexes were assayed following the reduction of substrate cyt *c*. The purified *bc*_1_ complexes were diluted to a final concentration of 0.1 μM and 1 μM based on the concentration of cyt *b* for *Btbc*_1_ and *Rsbc*_1_, respectively, in the B200 buffer (50mM Tris•HCl, pH 8.0, 0.01% *β*-DDM, 200 mM NaCl). The assay mixture contains 100 mM phosphate buffer, pH 7.4, 0.3 mM EDTA, and 80 μM cyt *c*, and Q_0_C_10_BrH_2_ at a final concentration of 5 μM. The addition of 3 μl of diluted *bc*_1_ solution initiates the reaction, which is recorded immediately following the cyt *c* reduction at 550 nm wavelength for 100 seconds in a two-beam Shimadzu UV-2250 PC spectrophotometer at 23°C. The amount of cyt *c* reduced over a given period of time was calculated using a millimolar extinction coefficient of 18.5 mM^−1^ cm^−1^.

To measure the effect of *bc*_1_ inhibitors, *bc*_1_ was pre-incubated at various concentrations of an inhibitor for 15 minutes prior to the measurement of its activity. The IC_50_ value was calculated by a least-squares procedure fitting the equation (Y = A_min_+(A_max_–A_min_)/(1+10^(X−logIC50)^) implemented in the commercial package Prism, where A_max_ and A_min_ are maximal and minimal activities, respectively. Although the chemical properties of Q_0_C_10_BrH_2_ are comparable to those of Q_0_C_10_H_2_, the former is a better substrate for the cyt *bc*_1_ complex isolated in detergent solution [22].

### Reaction Kinetics of *bc*_1_ in the Presence of Inhibitors

To measure the enzyme kinetics of cyt *bc*_1_ complex under inhibitory conditions, purified cyt *bc*_1_, either *Btbc*_1_ or *Rsbc*_1_, was assayed at different concentrations of substrates. When the Q_0_C_10_BrH_2_ concentration was varied (1 μM, 2 μM, 5 μM, 10 μM, 20 μM) the cyt *c* concentration was kept constant at 80 μM, whereas when the concentration of cyt *c* varied (1 μM, 2 μM, 4 μM, 8 μM, 12 μM, 16 μM) the Q_0_C_10_BrH_2_ concentration was kept constant at 50 μM. The reactions were initiated by adding 3 μl of diluted *bc*_1_ solution (0.1 μM for *Btbc*_1_ or 1.0 μM for *Rsbc*_1_) pre-incubated with various concentrations of inhibitors for 15 minutes. The time course of the absorbance change due to cyt *c* reduction was recorded continuously at 550 nm. Initial rates were determined from the slopes in the linear portion of cyt *c*_1_ reduction time course.

### Analysis of Cyt *bc*_1_ Spectra in the Presence of Inhibitors

For each run a solution of 1 ml bovine cyt *bc*_1_ at a cyt *b* concentration of 5 μM was fully reduced with addition of a tiny amount of sodium dithionite and its spectrum was obtained in the range of 520–600 nm. A specific inhibitor was added at various concentrations to the reduced *bc*_1_ complex and was scanned repeatedly until no changes were observed. All scans were stored digitally and difference spectra were produced by subtracting the corresponding spectrum of the inhibitor-free, fully reduced *bc*_1_ complex.

### Measurement of Cyt *b* and *c*_1_ Reduction Time Course in a Single Turnover Reaction

The enzyme was diluted to a final concentration of about 4 μM of cyt *c*_1_ in 1 ml of B200 buffer (50 mM Tris•HCl, pH 8.0, 0.01% *β*-DDM, 200 mM NaCl) and oxidized fully by adding a tiny amount of potassium ferricyanide. The spectrum of the fully oxidized enzyme in the range of 520–590 nm was stored. Inhibitors at various concentrations were introduced and incubated for 2 min followed by addition of the ubiquinol analog Q_0_C_10_BrH_2_ to a final concentration of 10 μM to start the reaction. Spectra were recorded at 20-second intervals starting immediately after mixing. After 800 seconds the enzyme was fully reduced by dithionite. The spectrum of the fully oxidized complex was subtracted from that at each time point and the amounts of reduced cyt *c*_1_ and *b* at a given time were calculated from the difference spectra at 552–540 nm and 560–576 nm, respectively.

### Molecular Docking of Pyrimorph to Cyt *b*

Coordinates for the receptor molecule were taken from the protein data bank (pdb) entry 1SQX, for the stigmatellin inhibited complex of *Btbc*_1_. The side chains of residues E271 and F274 were modeled as standard rotamers consistent with their positions in apo *Btbc*_1_. The ligand molecule, pyrimorph, was drawn and converted to a SMILES string using tools from the CADD Group [28]. The SMILES string was converted by the program Elbow [29] to 3D coordinates and energy minimized in GAMESS [30]. Both receptor and ligand molecules were converted to standard pdbqt files using mgltools [31]. For all docking runs, the acrylamide moiety (C7 = C8–C9 = O16) of pyrimorph was fixed in the *syn-periplanar* conformation because the alternative *anti-* conformation would bring the larger morpholino and pyridyl groups into close contact.

Two approaches to docking were taken: (1) as the most likely binding sites for inhibitors are the Q_P_ and the Q_N_ sites, docking attempts were made first at those known sites. (2) In a second set of runs, no prior knowledge of sites was imposed but the program Q-site-finder [32] was used to locate all potential binding sites and docking was carried out at all sensible locations. Docking pyrimorph to known and unknown inhibitor binding sites was performed using the program Autodock Vina [33].

## Results

### Pyrimorph Blocks the Mitochondrial Respiratory Chain by Targeting cyt *bc*_1_ Complex

To test whether pyrimorph inhibits fungal growth by interfering with the cellular energy metabolism pathway, in particular the mitochondrial respiratory chain, we isolated light mitochondrial fraction from the pathogenic fungus *P. capsici* and examined the ability of pyrimorph to inhibit various segments of the respiratory chain (Table 1). It is quite clear that pyrimorph has no effect on the activity of Complex I, as a concentration of pyrimorph as high as 16 μM was unable to inhibit NADH oxidation catalyzed by Complex I. By contrast, under the same conditions, pyrimorph inhibits 94.6% of Complex II activity. Most importantly, Complex III, the cyt *bc*_1_ complex, shows the highest sensitivity toward pyrimorph, with 95.3% inhibition even at 4 μM concentration.

**Table 1:** Inhibition of respiratory complexes I, II, and III by pyrimorph.

	Concentration of pyrimorph (μM)									
	0		4		8		12		16	
Complex	Activity*	% Inh	Activity	% Inh	Activity	% Inh	Activity	% Inh	Activity	% Inh
I (Inhibition of NADH oxidation)	51.8±2	0	52.7±3	−1.7	48.3±6	6.8	52.1±7	−0.5	49.8±1	3.8
II (Inhibition of DCPIP reduction)	112.7	0	83.6±4	25.8	58.3±6	48.3	25.1±7	77.7	6.8±1	94.6
III (Inhibition of cyt c reduction	27.24	0	1.29±1	95.3	–	–	–	–	–	–

*Millimolar extinction coefficients for oxidation NADH is 6.2 mM^−1 ^cm^−1^ [49], for DCPIP is 21 mM^−1 ^cm^−1^ [50], and for cyt *c* is 18.5 mM^−1 ^cm^−1^ [24].

**Figure 1:**
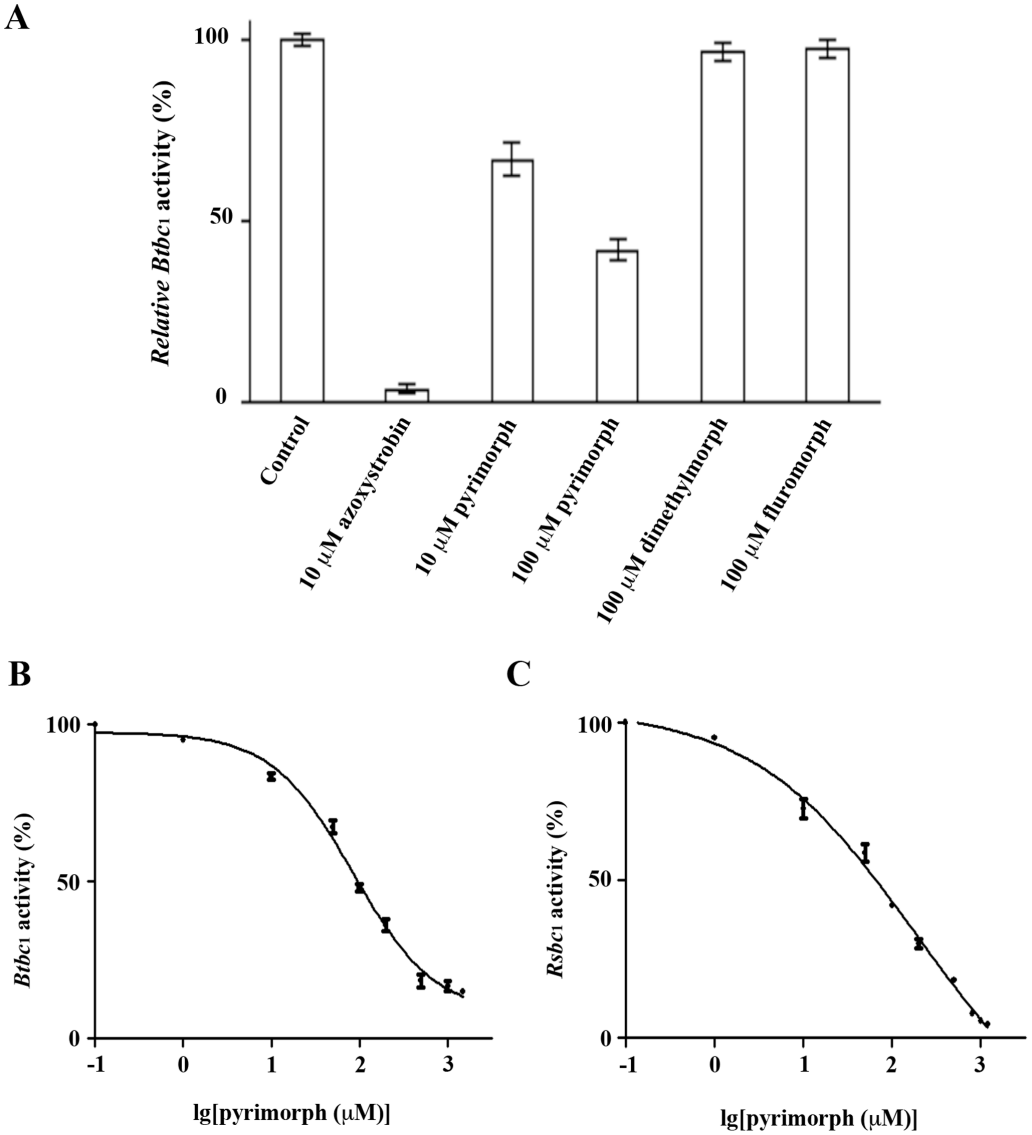
Inhibition of cyt *bc*_1_ by various inhibitors. (A) Inhibition of *Btbc*_1_ by several amide fungicides and azoxystrobin at indicated concentrations. The control is the activity of *bc*_1_ in the absence of inhibitor, which is set to 100%. (B) Concentration-dependent inhibition of *Btbc*_1_ by pyrimorph. (C) Concentration-dependent inhibition of *Rsbc*_1_ by pyrimorph.

Although the effects of pyrimorph on the mitochondrial respiratory chain of *P. capsici* were clearly demonstrated, such effects could be indirect. To ascertain that the target of pyrimorph is indeed the cyt *bc*_1_ complex, we used highly purified cyt *bc*_1_ from beef heart (*Bos taurus bc*_1_ or *Btbc*_1_) and assayed inhibition of its cyt *c* reductase activity by pyrimorph. The result was compared to the well-known anti-*bc*_1_ fungicide azoxystrobin and two other CAA-type fungicides, dimethomorph and flumorph. As shown in Fig. 1A, *Btbc*_1_ activities are reduced to 67% and 42% of the control, respectively, in the presence of 10 μM and 100 μM of pyrimorph. Azoxystrobin is able to inhibit *bc*_1_ activity by more than 95% at 10 μM concentration. However, the two CAA-type fungicides, dimethomorph and flumorph, displayed no activity at all against *Btbc*_1_. These results indicated that pyrimorph is different from other members of CAA-type fungicides and more importantly established that pyrimorph is indeed an inhibitor of the cyt *bc*_1_ complex, albeit a weak one.

We subsequently determined 50% inhibitory concentration (IC_50_) for pyrimorph against isolated cyt *bc*_1_ complexes from both bovine mitochondria and photosynthetic bacterium *R. sphaeroides* (*Rsbc*_1_). Pyrimorph is slightly more potent against *Rsbc*_1_, giving an IC_50_ value of 69.2 μM; it gives an IC_50_ of 85.0 μM for *Btbc*_1_ (Figs. 1B and 1C). As a comparison, the well-known *bc*_1_ inhibitor stigmatellin and azoxystrobin give IC_50_ values of 2.8 nM and 47.7 nM, respectively, for isolated *Btbc*_1_ by our measurement (data not shown) under the same assay conditions. Since *Rsbc*_1_ has only four subunits, it is therefore certain that pyrimorph targets the essential subunits of the *bc*_1_ complex.

### Effect of Pyrimorph Binding on Reduction of the Cyt *b* and *c*_1_ by Ubiquinol

**Figure 2:**
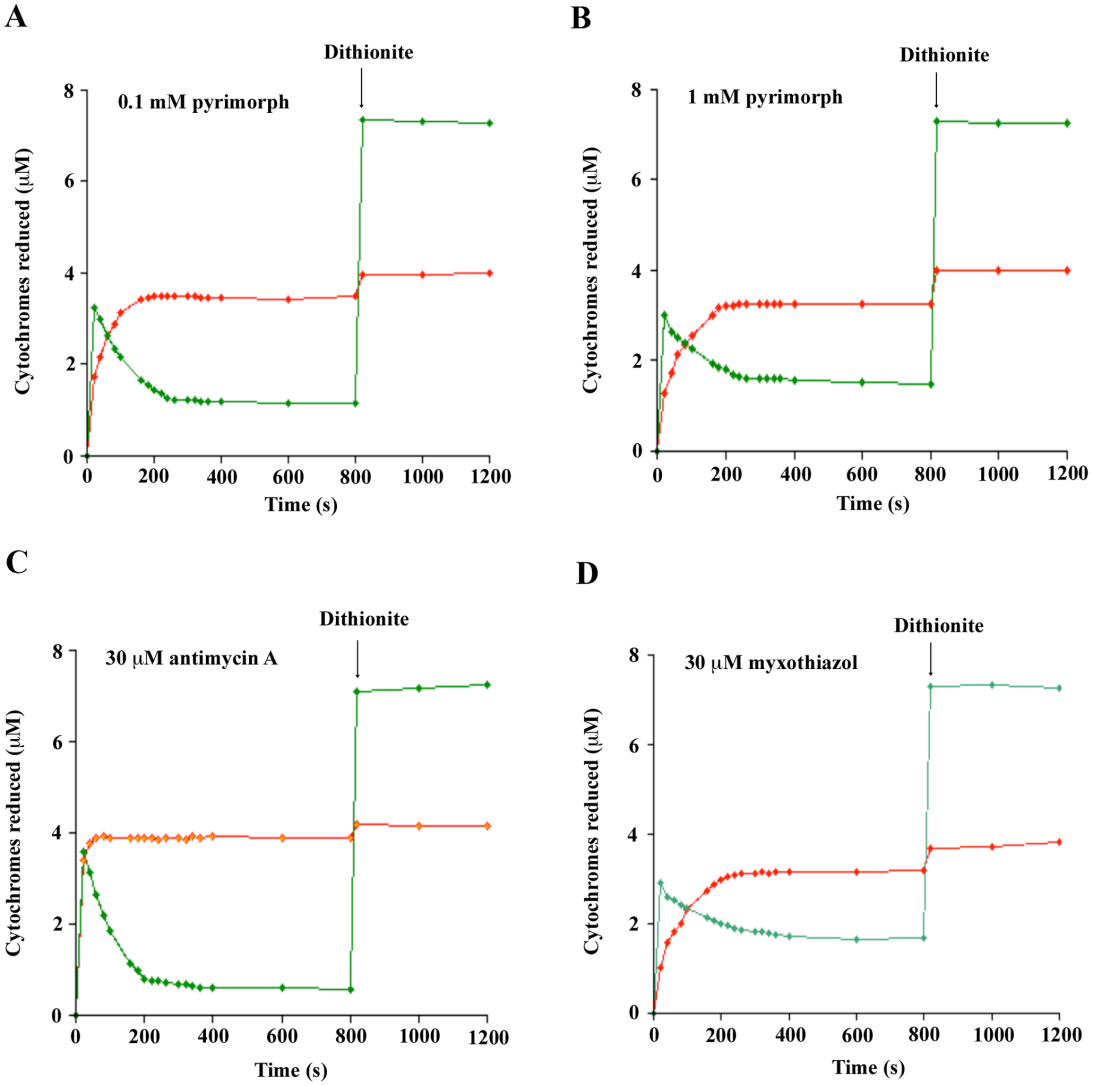
Influences of cyt *b* and *c*_1_ reduction by pyrimorph. Isolated *Btbc*_1_ was incubated with indicated inhibitors followed by single turn-over reaction initiated by addition of 10 μM Q_0_C_10_BrH_2_. The spectra were recorded immediately following the mixing and every 20 seconds thereafter. At the 800 second time point, a tiny amount of sodium dithionite was added to reduce both cyt *b* and *c*_1_. The amounts of reduced cyt *b* and *c*_1_ were calculated and plotted as a function of time. The green trace is the amount of cyt *b* reduced over time and the red one is the amount of cyt *c*_1_ reduced. (A) 100 μM pyrimorph, (B), 1000 μM pyrimorph, (C) 30 μM myxothiazol, and (D) 30 μM antimycin A.

Nearly all cyt *bc*_1_ inhibitors bind to the Q_N_ site, Q_P_ site or both [34]. It is known that binding of inhibitors produces various effects on spectra of cyt *b* and *c*_1_ heme groups, as well as on redox potential and conformation of the iron-sulfur protein [34]–[38]. These effects ultimately determine the rate and amount of cyt *b* or *c*_1_ reduced under equilibrium conditions and can be exploited to compare modes of action of different inhibitors [39], distinguishing for example a Q_N_ site inhibitor from a Q_P_ site one. Starting with a completely oxidized enzyme, *Btbc*_1_ was mixed with substrate Q_0_C_10_BrH_2_ in the presence of pyrimorph at two different concentrations (0.1 mM and 1.0 mM); no cyt *c* was used as the terminal electron acceptor. The amount of cyt *b* including *b*_L_ and *b*_H_ hemes and cyt *c*_1_ reduced as a function of time was recorded (Figs. 2A and 2B). The results were compared with those produced by Q_N_ site inhibitor antimycin A (Fig. 2C) and by Q_P_ site inhibitor myxothiazol (Fig. 2D).

In the absence of the high-potential electron acceptor cyt *c*, only a single enzymatic turnover at the Q_P_ site is possible when the Q_N_ site inhibitor antimycin A is bound. Under such conditions, both cyt *b* and *c*_1_ were rapidly reduced reaching maximal reduction of nearly half of the *b*-type hemes and all of the *c*-type heme perhaps even before the first measurement was recorded (Fig. 2C). Once reaching maximal reduction, cyt *b* began non-enzymatic oxidation rather rapidly, whereas the redox state of cyt *c*_1_ remained unchanged. When the Q_P_ site is occupied by an inhibitor such as myxothiazol, not a single turnover is possible (Fig. 2D). The initial rapid reduction of cyt *b* was most likely via the revere reaction at the Q_N_ site and cyt *c*_1_ reduction was entirely non-enzymatic. Thus, the rate of cyt *c*_1_ reduction and that of cyt *b* re-oxidation can be employed to determine which site an inhibitor targets.

Clearly, the reduction behavior of cytochromes *b* and *c*_1_ induced by pyrimorph distinguishes it from that induced by antimycin A (Figs. 2A, 2B and 2C), demonstrating that pyrimorph does not target the Q_N_ site. By contrast, the time courses of cyt *b* and *c*_1_ reduction in the presence of pyrimorph or myxothiazol resemble each other (Figs. 2A, 2B, and 2D), except that the latter inhibitor takes a longer time for cyt *c*_1_ to be maximally reduced than pyrimorph does. This result puts pyrimorph into the category of a Q_P_-site inhibitor by either directly or indirectly competing with ubiquinol for the Q_P_ site.

### Spectral Analyses Suggest the Binding of Pyrimorph near Q_P_ Site

Single turnover experiments suggested that pyrimorph acts in a fashion similar to that of Q_P_ site inhibitors. It was thus necessary to determine how pyrimorph interferes with *bc*_1_ function at the Q_P_ site. Since spectral changes, especially red shifts in the α- and β-band caused by binding of inhibitors to reduced *bc*_1_, were successfully used to deduce information about their binding interactions [37], [40], a similar approach was taken for pyrimorph. By comparing the spectra of reduced *bc*_1_ bound with pyrimorph to those obtained with known Q_P_ or Q_N_ site inhibitors such as myxothiazol or antimycin A, we hoped to gain insight into the binding interactions and location.

**Figure 3:**
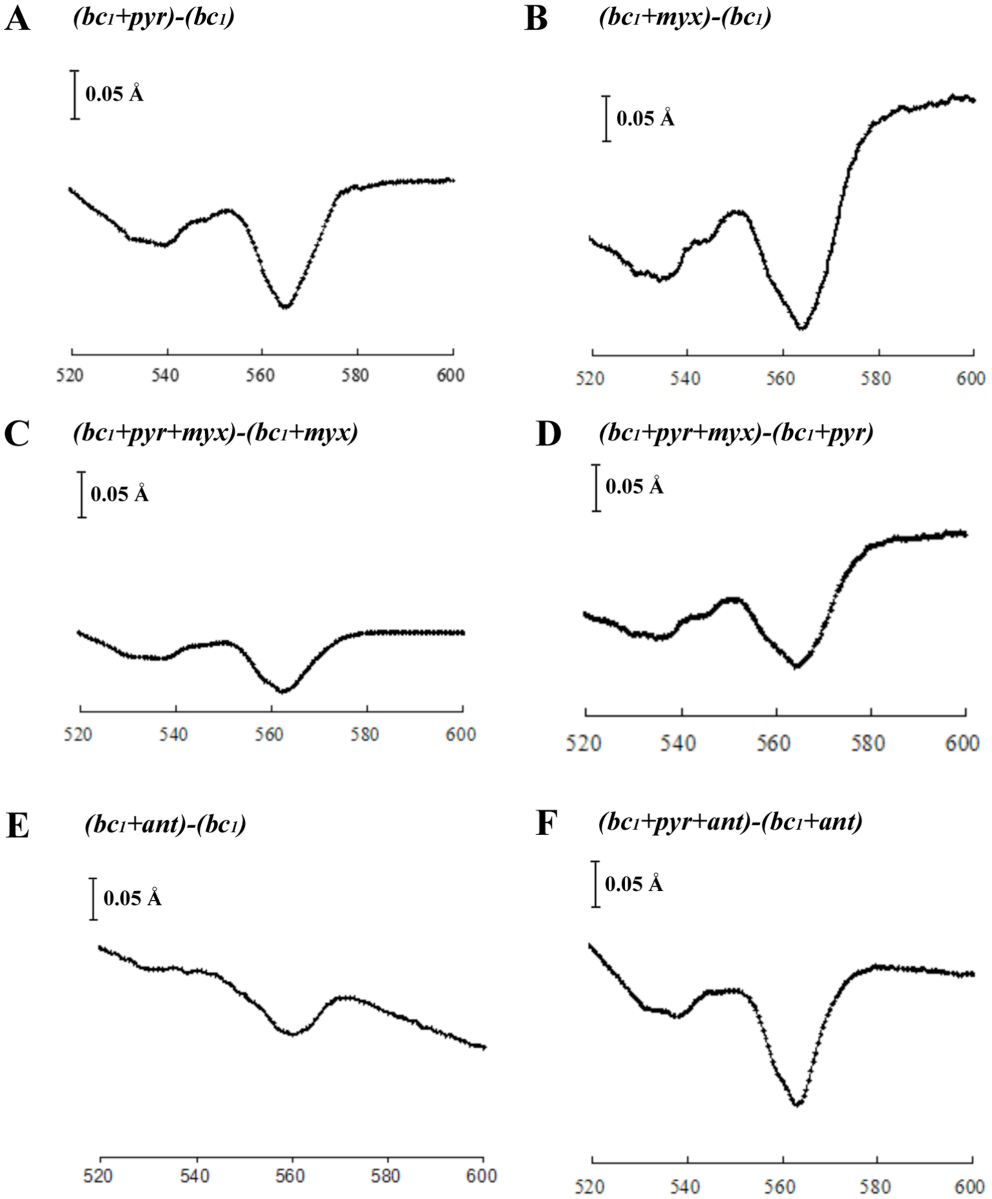
Difference spectra of inhibitors and inhibitors combinations to reduced Btbc1. All spectra were recorded with purified *Btbc*_1_ at a concentration of 5 μM of cyt *b* with the concentrations of inhibitor as indicated. Prior to spectral scan, the *bc*_1_ complex was reduced by addition of dithionite. (A) Spectrum of reduced *Btbc*_1_ in the presence of 1 mM pyrimorph (pyr) minus that of reduced *Btbc*_1_ alone. (B) Spectrum of reduced *Btbc*_1_ in the presence of 10 μM myxothiazol (myx) minus that of reduced *Btbc*_1_ alone. (C) and (D) The spectrum of reduced *Btbc*_1_ in equilibration with 1 mM pyrimorph followed by addition of 10 μM myxothiazol minus spectrum of reduced *Btbc*_1_ in the presence of 10 μM myxothiazol or 1 mM pyrimorph, respectively. (E) Spectrum of reduced *Btbc*_1_ in the presence of 10 μM antimycin A (ant) minus spectrum of reduced *Btbc*_1_ alone. (F) Spectrum of reduced *Btbc*_1_ after equilibration with 1 mM pyrimorph and 10 μM antimycin A in sequence minus spectrum of reduced *Btbc*_1_ in the presence of antimycin A.

Binding of pyrimorph causes the spectrum to red shift, as the difference spectrum [(*bc*_1_+pyr)-*bc*_1_] shows a trough centered around 565 nm (Fig. 3A), which is an indication that binding of pyrimorph affects cyt *b* hemes. This spectrum was compared to spectra with bound Q_P_ site inhibitor myxothiazol [(*bc*_1_+myx)-*bc*_1_] and Q_N_ site inhibitor antimycin A [(*bc*_1_+ant)-*bc*_1_], respectively (Figs. 3B and 3E). At a first glance, it seems that the spectral change due to pyrimorph binding resembles that caused by myxothiazol binding, despite considerable differences (see below), indicating that pyrimorph binds closer to the *b*_L_ heme or the Q_P_ site. Indeed, binding pyrimorph to *bc*_1_ does not seem to interfere with subsequent binding of antimycin A, as the difference spectrum of [(*bc*_1_+pyr+ant) – (*bc*_1_+ant)] (Fig. 3F) looks almost identical to [(*bc*_1_+pyr)-(*bc*_1_)] (Fig. 3A). This experiment confirms that pyrimorph does not target the Q_N_ site.

However, binding of pyrimorph to *bc*_1_ does affect subsequent binding of myxothiazol and *vise versa*, because the difference spectrum [(*bc*_1_+pyr+myx) – (*bc*_1_+myx)] (Fig. 3C) does not look like that of [(*bc*_1_+pyr)-(*bc*_1_)] (Fig. 3A), nor does the difference spectrum [(*bc*_1_+pyr+myx) – (*bc*_1_+pyr)] (Fig. 3D) resemble that of [(*bc*_1_+myx)-(*bc*_1_)] (Fig. 3B). These spectra indicate the possibility that both inhibitors can co-exist near the Q_P_ pocket and influence each other. Since the binding of myxothiazol to the Q_P_ pocket is well established, the experiment further suggests that pyrimorph may have a different binding mode from that of myxothiazol.

### Inhibitory Kinetics of Cyt *bc*_1_ Suggests the Mode of Pyrimorph Action

**Figure 4:**
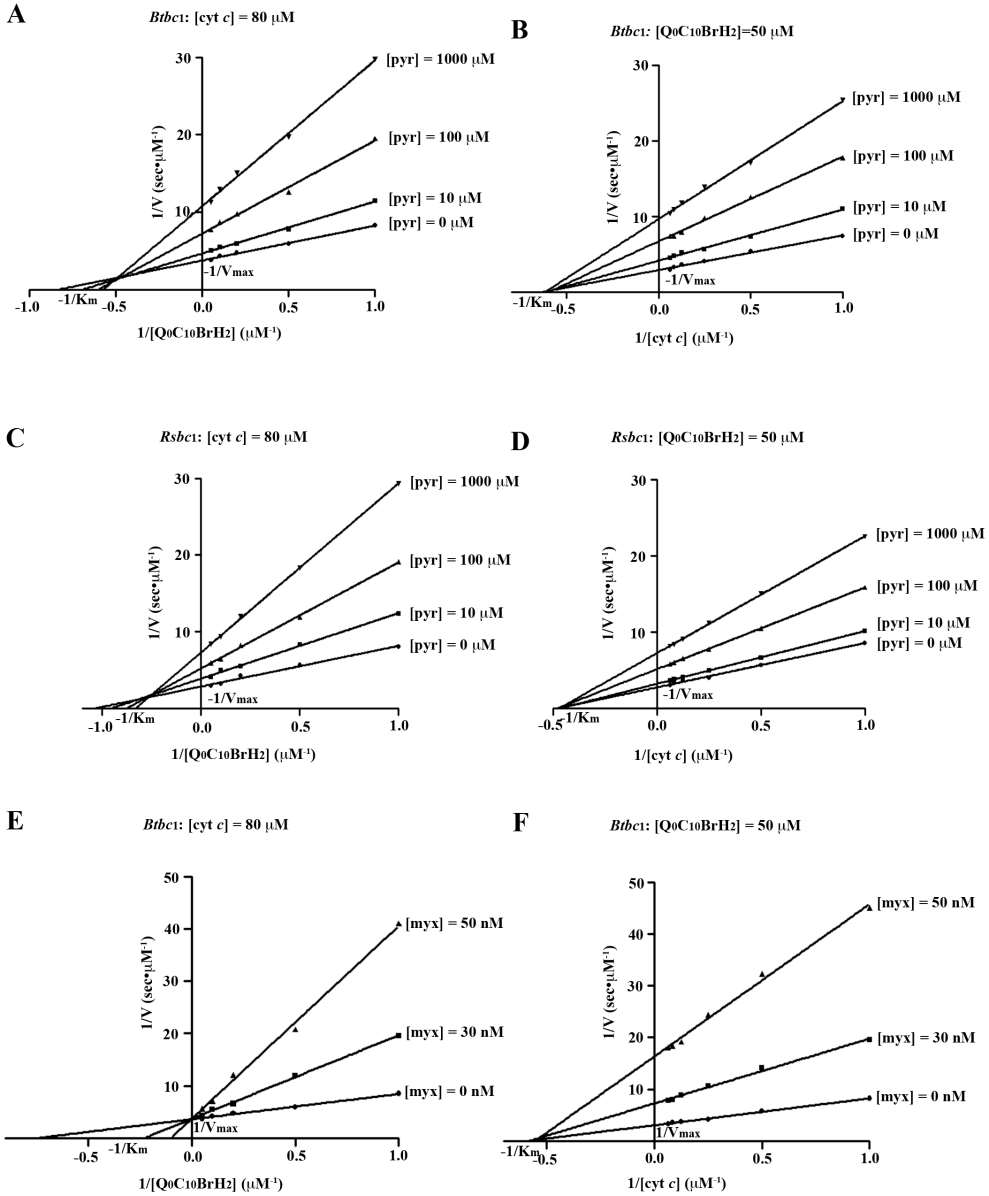
Double-reciprocal (Lineweaver-Burk) plots for *bc*_1_ inhibition. Four different concentrations of 0, 10, 100 and 1000 μM were used for pyrimorph and three, 0, 30 and 50 nM were used for myxothiazol. Each point represents a mean value of at least 3 independent experimental measurements. (A) Inhibition of *Btbc*_1_ by pyrimorph with variations in concentration of Q_0_C_10_BrH_2_. (B) Inhibition of *Btbc*_1_ by pyrimorph with variations in concentration of cyt *c*. (C) Inhibition of *Rsbc*_1_ by pyrimorph with variations in concentration of Q_0_C_10_BrH_2_. (D) Inhibition of *Rsbc*_1_ by pyrimorph with variations in concentration of cyt *c*. (E) Inhibition of *Btbc*_1_ by myxothiazol with variations in concentration of Q_0_C_10_BrH_2_. (F) Inhibition of *Btbc*_1_ by myxothiazol with variations in concentration of cyt *c*.

The possibility that pyrimorph has a different mode of action is of particular interest in light of our extensive knowledge on the development of resistance to existing inhibitors. To further probe the mechanism of pyrimorph’s action, we investigated the kinetic properties of *bc*_1_ function under pseudo first-order reaction conditions by measuring its activity with respect to changes in concentration of either substrates Q_0_C_10_BrH_2_ or cyt *c* in the presence of different amount of pyrimorph, allowing double reciprocal or Lineweaver-Burk plots to reveal the relationship between 1/V and 1/[S]. The measurements were done for both *Btbc*_1_ and *Rsbc*_1_, revealing nearly identical kinetic behavior (Fig. 4). As shown in Figs. 4A and 4C, in the presence of a constant 80 μM cyt *c* and with increasing concentrations of Q_0_C_10_BrH_2_, both *K*_m_ and *V*_max_ are altered as is the *K*_m/_ *V*_max_ ratio. Expectedly, as the concentration of pyrimorph increases, the *V*_max_ decreases; at a constant pyrimorph concentration, the reciprocal enzyme activity 1/V has a positive slope with respect to 1/[S]. However, the *K*_m_ value for the substrate quinol changed, falling between competitive (Fig. 4E) and non-competitive (Fig. 4F) inhibitions. Thus, pyrimorph falls into the category of a mixed-type, noncompetitive inhibitor with respect to the substrate ubiquinol, suggesting that it competes, both directly and indirectly, with ubiquinol to occupy the Q_P_ site.

At a constant 50 μM Q_0_C_10_BrH_2_ concentration and with varying concentrations of cyt *c*, double-reciprocal plots show that x-intercepts remain the same with or without pyrimorph (Figs. 4B and 4D), suggesting that the apparent *K_m_* for substrate cyt *c* remains unchanged. Thus, pyrimorph is a noncompetitive inhibitor with respect to cyt *c*. As a control, we performed the same experiments with *Btbc*_1_ using the classic Q_P_-site inhibitor myxothiazol, showing that myxothiazol is a competitive inhibitor for the substrate quinol but a non-competitive inhibitor for cyt *c* (Figs. 4E and 4F).

### Docking of Pyrimorph to cyt *b* Subunit

**Figure 5:**
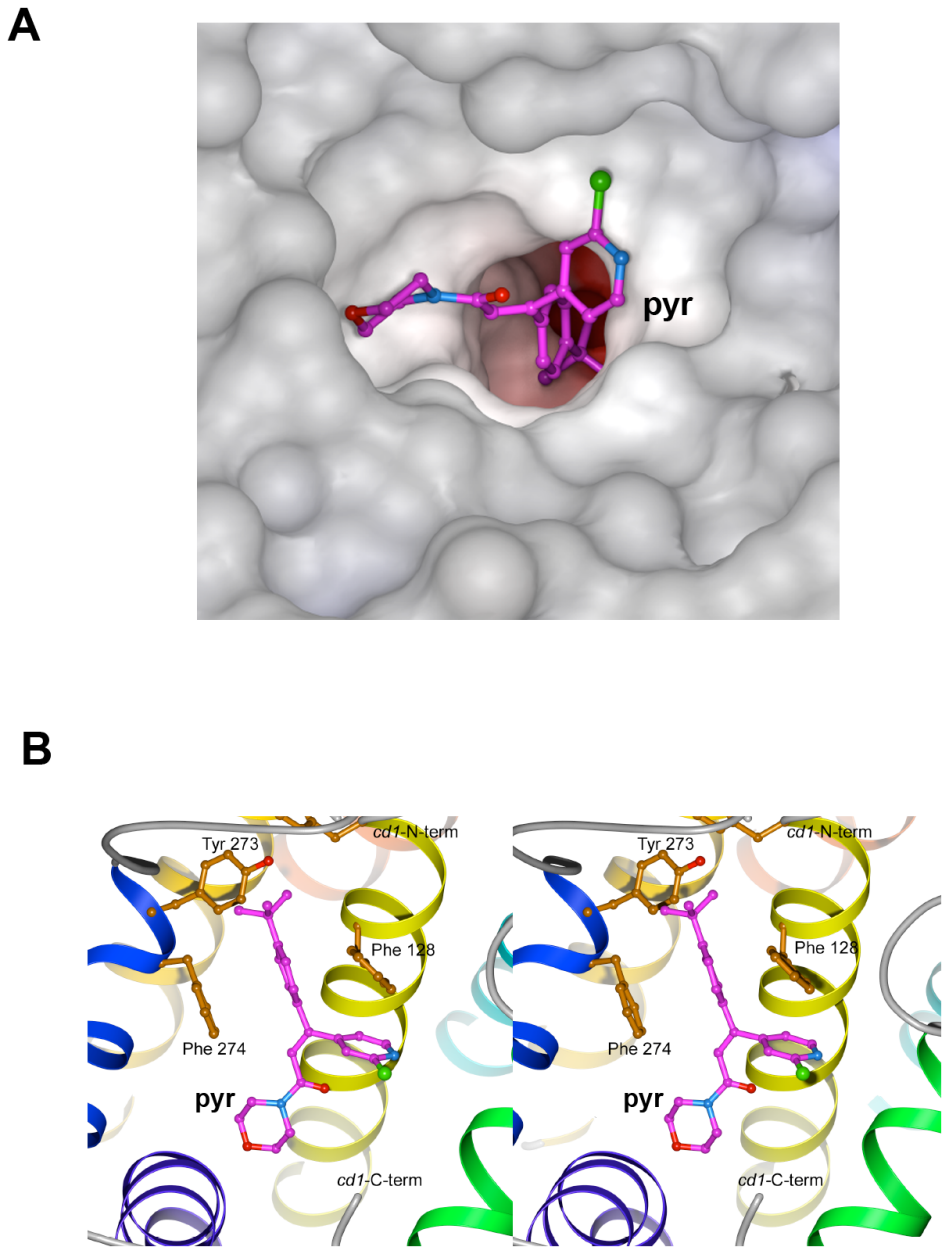
Molecular docking showing the possible binding site and interaction of pyrimorph in the cyt *b* subunit. (A) Molecular surface of the cyt *b* subunit is given, showing the access portal leading to the Q_P_ site, which is blocked by the docked pyrimorph in a ball-and-stick model with atoms of carbon in magenta, nitrogen in blue, oxygen in red and chlorine in green. (B) Stereoscopic pair showing the detailed interactions between residues in the cyt *b* subunit with the docked pyrimorph molecule.

Docking of pyrimorph to known inhibitor-binding sites in the cyt *b* subunit of *Btbc*_1_ were performed with Autodock Vina and resulted in top solutions at the Q_P_ site with a binding free energy of −9.7 kcal/mol and −9.2 kcal/mol at the Q_N_ site, representing a 2.3-fold difference in binding affinity between the two sites. These energy values can be compared with binding of other known *bc*_1_ inhibitors such as stigmatellin, giving rise to a binding free energy of −10.5 kcal/mol. Potential inhibitor binding sites outside the known active sites were searched by Q-site-finder and the top 20 sites suggested (which included the Q_P_ and Q_N_ site) were subjected to extensive docking trials using Autodock Vina but no new locations showing improved affinity over the classic sites were identified. The Q_P_ site showed the highest binding affinity to pyrimorph. Unlike traditional inhibitors, pyrimorph does not enter the Q_P_ site, but rather blocks the entrance or portal to the quinol oxidation site (Fig. 5A). While its morpholino and 4-(2-chloro pyridyl) moieties stay in the central cavity of the *bc*_1_ dimer, its 4-(*tert*-butyl) phenyl group enters the access portal, where it is stabilized by the aromatic side chain of F274 and partially by F128. The latter primarily interacts with the pyridyl moiety via aromatic-aromatic (Ar-Ar) interactions. The *tert*-butyl group that penetrates into the Q_P_ site is flanked by the residues Y273, Y131 and P270, establishing beneficial van-der-Waals contacts.

## Discussion

Resistance to cyt *bc*_1_ inhibitors has been extensively investigated, revealing a wide variety of underlying mechanisms including target site mutations [41], [42], activation of alternative oxidase pathways [43], [44], altered metabolic degradation [45], reduced uptake and increased efflux [46], [47]. By far, target site mutation is the most prevalent form of resistance that develops against *bc*_1_ inhibitors. Thus, extensive research has been focusing on how to overcome resistance caused by target site mutations. Finding inhibitors that target alternative sites seems to be an attractive strategy.

### Pyrimorph is a Multi-target Fungicide Displaying Inhibitory Activity against Cyt *bc*_1_

Pyrimorph is a fungicide containing a carboxylic acid amide (CAA) moiety and was shown to be cross-resistant with other CAA fungicides such as mandipropamid, dimethomorph and flumorph [21], suggesting the possibility that pyrimorph may function in a manner similar to that of other CAA-type fungicides such as mandipropamid for which the mode of action was established by inhibiting cellulose synthase 3 or CesA3 [19]. In a recent publication [21], pyrimorph-resistant isolates of *P. capsici* were selected in the presence of the inhibitor and the three most resistant strains share a common mutation (Q1077K) in the CesA3 gene, which is different from the one (G1104V) selected for mandipropamid resistance [19]. However, it remains to be seen whether transfer of the resistant allele to the sensitive parental strain would make the latter pyrimorph-resistant. So far there is no direct evidence from *in vitro* biochemical experiments that shows at the protein level the inhibition of CesA3 by pyrimorph.

In the current study, we followed up on the previous observation that pyrimorph may act on the cellular respiratory chain of pathogenic fungi [20]. We showed that pyrimorph is able to suppress the respiratory chain function at 4 μM concentration by inhibiting the activity of Complex III in isolated mitochondria of *P. capsici* mycelia (Table 1). We further showed conclusively that pyrimorph inhibits purified mitochondrial as well as bacterial *bc*_1_ complexes with IC_50_ values at sub-millimolar range (Fig. 1). By contrast, two other CAA-type inhibitors, dimethomorph and flumorph, displayed no inhibitory activity against *bc*_1_ complex (Fig. 1A).

It did not escape our notice that cyt *bc*_1_ in light mitochondrial fraction isolated from *P. capsici* mycelia appears to be more sensitive to pyrimorph than purified bovine or bacterial *bc*_1_ complexes, suggesting the following possibilities: (1) Direct comparison between results of two very different assays is not a fair comparison, because in isolated light mitochondrial fraction the estimation of cyt *bc*_1_ concentration is difficult in the presence of many different proteins. However, the inhibitory concentrations or IC values are directly related to the amount of enzyme in the assay solution. So the lower IC value could be due to a lower concentration of *bc*_1_ in the assay conditions. (2) Being a hydrophobic compound, pyrimorph may preferentially partition into the lipid bilayer of mitochondrial membranes, leading to a higher local concentration and in turn to the apparent 95% inhibition at 4 μM concentration (Table 1, Fig. 1). (3) Conversely, the presence of detergent (micelles) in the solution of purified *bc*_1_ complex might lower the effective concentration of pyrimorph. This scenario is less likely, as the concentration of β-DDM in our assay buffer is barely above one critical micelle concentration (CMC). (4) The cyt *bc*_1_ of *P. capcisi* is more sensitive to pyrimorph than either bovine or bacterial *bc*_1_. However, we note that the purified bacterial complex is more sensitive to pyrimorph than bovine *bc*_1_ but only by a factor of 1.2. The difference might simply be a reflection of changes in the sequences and we do observe that bacterial *bc*_1_ exhibits slightly higher similarity to fungal than bovine mitochondrial cyt *b*.

### Pyrimorph Likely Acts Near but not at the Q_P_ Site

The fact that pyrimorph inhibits both 11-subunit *Btbc*_1_ and 4-subunit *Rsbc*_1_ demonstrates that the inhibitor acts on cyt *b*, cyt *c*_1_ or ISP subunits of the complex; it does not inhibit mitochondrial *bc*_1_ function through binding to the so-called supernumerary subunits (Figs. 1B and 1C). The two potential sites for pyrimorph binding are Q_N_ and Q_P_ in the cyt *b* subunit and so far all experimental evidence suggests a binding site near the Q_P_ site: (1) Single turn-over experiments show the reduction rate of cyt *c*_1_ and re-oxidation rate of cyt *b* in the presence of two different concentrations of pyrimorph (Figs. 2A and 2B) are very similar to those in the presence of the Q_P_ site inhibitor myxothiazol (Fig. 2D), but are drastically different from those in the presence of the Q_N_ site inhibitor antimycin A (Fig. 2C). (2) Difference spectra of reduced cyt *bc*_1_ also provided strong evidence that pyrimorph targets the Q_P_ site (Fig. 3), because the difference spectrum of [(*bc*_1_+pyr)-(*bc*_1_)] (Fig. 3A) resembles that of [(*bc*_1_+myx)-(*bc*_1_)] (Fig. 3B) but not that of [(*bc*_1_+ant)-(*bc*_1_)] (Fig. 3E).

While the analysis of the difference spectra points to the Q_P_ pocket as the target site, it also suggests that pyrimorph has a non-overlapping binding site with the classic Q_P_ site inhibitor myxothiazol (Fig. 3). Indeed, double-reciprocal or Lineweaver-Burk plots of *bc*_1_ activity showed that pyrimorph acts as a mixed-type, non-competitive inhibitor with respect to the substrate ubiquinol (Figs. 4A and 4C), suggesting that pyrimorph may act both competitively and non-competitively for the substrate ubiquinol (Fig. 4E). Mechanistically, it means that pyrimorph is capable of modulating the binding of the substrate ubiquinol without directly competing with it at the active site, which categorizes it as a mixed-type, non-competitive inhibitor [48].

Unlike classic Q_P_ site inhibitors that compete directly with substrate ubiquinol for interactions with the same set of residues in the Q_P_ site, pyrimorph rather seems to block the portal to the Q_P_ site, through which the substrate ubiquinol has to pass to contact ISP. Simultaneously, a good portion of pyrimorph is held outside the substrate-binding pocket by hydrophobic forces. Consequently, ubiquinol has to actively displace pyrimorph from the entrance in order to gain access to the Q_P_ site as in the case of a competitive inhibitor. On the other hand, as pyrimorph has the ability to adhere well to the lipophilic sides of the portal that leads to the Q_P_ site of cyt *b*, it may stay close and possibly interfere with the necessary motion of the cd1/cd2 helix and with the release of ubiquinone, displaying inhibitory activities more characteristic of non-competitive inhibitors. This picture is entirely consistent with biochemical and spectral characterizations of pyrimorph, qualifying it as a mixed-type, non-competitive inhibitor.

Molecular modeling of other CAA-type fungicides such as dimethomorph indicate a very similar binding position and orientation to the Q_P_ site but with a significantly lower free energy for binding, consistent with the observation that both dimethomorph and flumorph are not *bc*_1_ inhibitors (Fig. 1A). Since both dimethomorph and flumorph structurally resemble pyrimorph, it is clear that the shape of pyrimorph is not a dominant factor for its ability to bind *bc*_1_. At the very least the binding of pyrimorph has sparked ideas for a dual approach to inhibitor design: (1) on the side that binds to the Q_P_ entrance, modifications could significantly increase the affinity, making it a better competitive inhibitor. (2) Improvements in hydrophobicity or geometric factors on the side that stays outside the Q_P_ pocket, the inhibitor could enhance its non-competitive properties. Should it be possible to design a dual type inhibitor, fungal resistance may be stalled for an extended period. Suggestions for improvements might include modifications of the *tert*-butyl group to include polar groups (hydroxy methyl, methoxy methyl, etc.) that are within the reach of E271 (both sidechain and backbone amide) as well as sterically demanding aromatic groups that may take advantage of the large, aromatic cavity of the Q_P_ site. On the side that stays outside of the Q_P_ pocket, variations of saturated and aromatic ring systems seem likely to improve binding properties, as it appears that the morpholino group and the chloro-pyridyl group can change places with minimal change in binding energy.
